# The Clinic and Management of a Prolapsed Gravid Uterus Incarcerated Through the Anal Orifice in the Resource‐Limited Setting: A Case Report

**DOI:** 10.1155/crog/1169916

**Published:** 2026-02-27

**Authors:** Matabishi Bandeke Destin, Mongwa Mbikilile Justin, Heritier Balirwa, Byabene Gloire, Mukanire Ntakwinja, Alumeti Munyali Désiré

**Affiliations:** ^1^ Department of Surgery, Panzi General Hospital, Medicine Faculty and Public Health, Université Évangélique en Afrique, Bukavu, Congo; ^2^ Department of Obstetrics and Gynecology, Panzi General Hospital, Medicine Faculty and Public Health, Université Évangélique en Afrique, Bukavu, Congo

**Keywords:** incarcerated gravid uterus, limited resources, surgical clinic and treatment, uterine prolapse

## Abstract

Prolapse of the gravid uterus, which cannot be spontaneously reduced through the anal orifice, is extremely rare. It is considered to be incarcerated and constitutes a clinical and therapeutic emergency, as it endangers fetal and maternal life. However, few cases have been reported in the literature. Because of the rarity of these prolapses of the gravid uterus and their clinical presentation, especially in young women in rural areas with armed conflicts, in the form of a mass protruding from the anal canal with a reduction in the volume of the abdomen, the clinical diagnosis provides guidance, and an ultrasound gives the age of the pregnancy. However, reduction by laparotomy under general anesthesia can be a therapeutic alternative in resource‐limited settings. In this study, we report on the clinic and management of a prolapsed gravid uterus incarcerated through the anal orifice in a 24‐year‐old woman in a resource‐limited setting.

## 1. Introduction

The prevalence of an incarcerated gravid uterus combined with rectal prolapse is scarce, and only a few cases have been reported previously, including a third in 2021 in Egypt [1]. Uterine prolapse is a form of pelvic organ prolapse in which the uterus, cervix, and upper vagina protrude into the vaginal canal more often than not [2]. However, this gravid uterus can rarely pass through the anal canal and cause complications that endanger the lives of both the mother and the fetus. The mechanism most often involves the role of biomechanical forces and alterations in the anatomical structures that support and/or separate the rectum and the pregnant uterus [3].

Uterine retroversion is a common and normal alteration in women, with a 15% incidence rate. Between 14 and 16 weeks of pregnancy, the uterus naturally pivots to an anteverted posture, allowing the expanding uterus to extend into the abdomen. However, if the uterus fails to antevert and move up into the abdominal cavity, it becomes wedged in the hollow of the sacrum and becomes incarcerated [4, 5]. This study is aimed at reporting on the clinic and management of a prolapsed gravid uterus incarcerated through the anal orifice in the resource‐limited setting.

## 2. Case Presentation

The patient is a 24‐year‐old woman residing in a rural area of the eastern Democratic Republic of the Congo. She presents with a 7‐year history of recurrent anal prolapse episodes, previously managed by manual reduction at home. Her obstetric history includes a gravidity of 5, parity of 2, two living children, and two spontaneous abortions. Her last menstrual period was recorded on June 27, 2024. She sought medical attention following the sudden appearance of a painful anal mass, which had previously been reducible but had become irreducible despite repeated attempts at home. This episode was accompanied by a noticeable decrease in abdominal volume, occurring approximately 2 h before presentation, during an effort to defecate. A second attempt at manual reduction was made at a local health clinic, but it failed, prompting a referral to Salamabila General Hospital for additional treatment.

On physical assessment, an expression of pain marked the general condition, although her vital signs remained within normal limits. The thoracic examination was normal. However, a reduction in abdominal volume was noted, and the gravid uterus was not palpable. Gynecological and obstetrical examination revealed vulvar staining with mucous and bloody discharge. A large, protruding, reddish mass was observed, obstructing access to both the vaginal and rectal canals, rendering digital assessment impossible. The mass was nonreducible and covered with patches of fecal material (Figures 1a, 1b, and 1c). It was rounded, with a smooth and regular surface, and protruded through the anal canal. The anal orifice was present and patent, permitting palpation of an anteriorly located mass.

Figure 1(a) Incarcerated prolapsed uterus in a pregnant patient, emerging through the anal canal, with prior urinary catheter placement. (b) Image after failed manual reduction by perineal approach. (c) Profile view of an incarcerated gravid uterus at 21 weeks′ amenorrhea emerging through the anal canal.

**Figure:**
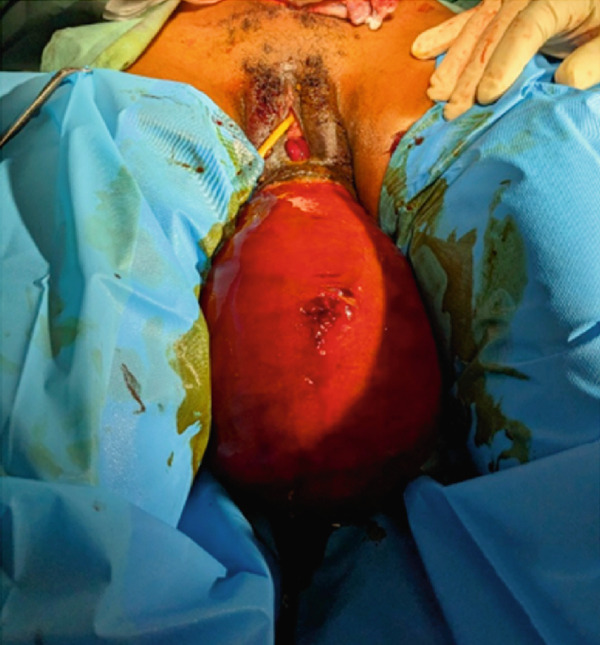
(a)

**Figure:**
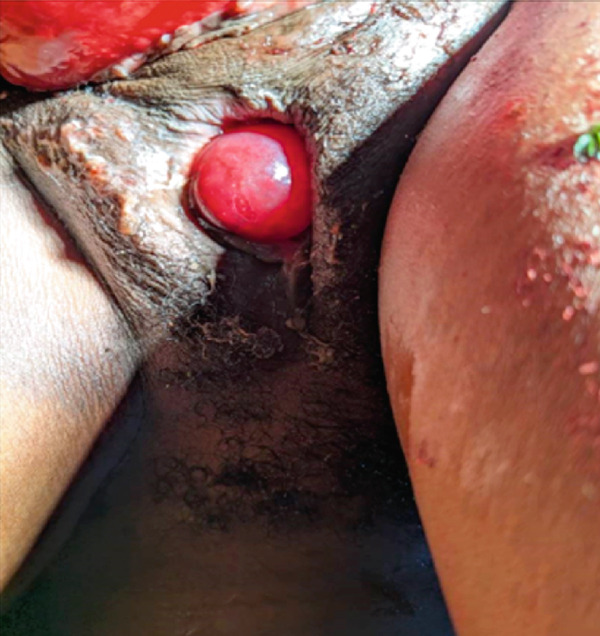
(b)

**Figure:**
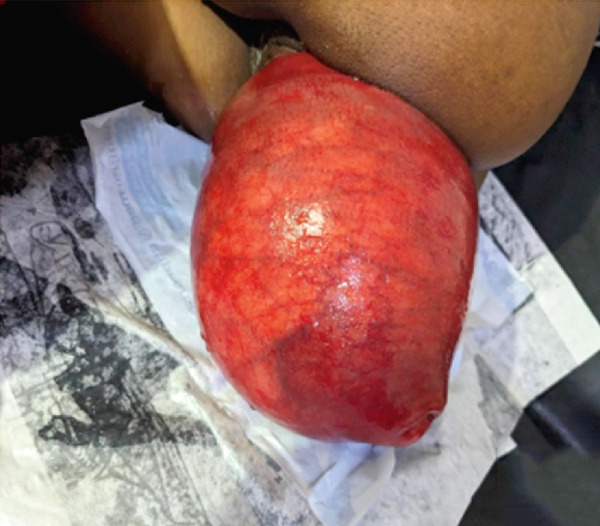
(c)

A diagnosis of incarcerated prolapse of the gravid uterus through the anal orifice was established. The pregnancy test returned positive, the complete blood count was within normal parameters, and no pelvic or perineal ultrasound was performed. An attempt was made to reduce the prolapsed uterus using sedated manual maneuvers, but this proved unsuccessful. Consequently, an exploratory laparotomy was indicated and performed. Intraoperative findings revealed an empty abdominopelvic cavity, with no evidence of the gravid uterus in its expected anatomical location. Following reduction via the abdominal route, areas of necrosis were identified on a segment of the rectum. Further exploration, involving systematic palpation of the pelvic organs, confirmed that the uterus had been externalized through the anal orifice, outside the abdominal cavity.

Perineal reduction combined with progressive abdominal traction returned the uterus to the abdominal cavity, and the necrotic rectal segment was resected, followed by terminal colo‐anal anastomosis with Vicryl 2/0 wire. An anal cerclage with Vicryl 1 thread was also performed to avoid a probable recurrence; meanwhile, plane‐by‐plane closure of the abdominal wall was carried out. Antibiotic therapy consisting of Augmentin 3 times 1.25 g and Flagyl 3 times 500 mg was instituted for 21 days, and a semiliquid diet was introduced on Postoperative Day 4. A laxative was prescribed on Postoperative Day 5. The postoperative course was uncomplicated, and intestinal transit was restored by Postoperative Day 2. Postoperative fetal heartbeat auscultation was used to monitor the pregnancy, and a transfer to a hospital where she was able to perform the ultrasound was arranged.

An obstetric and abdominopelvic ultrasound performed at the referral hospital demonstrated normal progression of the pregnancy. After reaching 38 weeks of amenorrhea, the patient returned to our facility expressing a desire to deliver via cesarean section. The procedure was carried out successfully, resulting in favorable extrauterine adaptation of the newborn and an uneventful maternal recovery.

## 3. Discussion

This case report was aimed at reporting on the clinic and management of a prolapsed gravid uterus incarcerated through the anal orifice in the resource‐limited setting, and this study suggests that the sign was observed in which the emergence of a mass at the level of the anal orifice was irreducible and painful in a woman who was diagnosed with a progressive pregnancy associated with a sudden decrease in abdominal volume; one must also think of a prolapse of the gravid uterus incarcerated. In resource‐constrained circumstances where ultrasonography is not possible, a pregnancy test may confirm the presence of a pregnancy; nevertheless, an exploratory laparotomy not only verifies the diagnosis but also may allow for safe reduction for the woman and fetus.

### 3.1. Clinical Data

It has been reported in the literature that rectal prolapse is a rare condition affecting approximately 0.5% of the population. It generally presents in older women, and often long‐standing constipation due to pelvic floor disorders or medication is the sign associated with prolapse [6]. Our patient was young, and the urgency of the prolapse associated with pain had not allowed for the establishment of this sign of constipation, which explains the complexity of our case. Given that certain signs may have been present but were difficult to assess, as maternal–fetal well‐being was the emergency, this is likely the reason.

The coexistence of a prolapsed, incarcerated gravid uterus within a rectal prolapse is exceedingly rare, with only a few cases documented in the literature to date [7]. The first reported case was published by Hassanin et al. [8] in 2016, and our case adds to this limited body of evidence, highlighting the diagnostic challenges encountered in resource‐limited settings affected by armed conflict, where imaging modalities are often unavailable.

Despite a thorough clinical assessment, we believe that imaging plays a critical role in both the diagnosis and maternal–fetal prognosis. In our case, perineal and abdominal ultrasonography would have been of vital importance. Dong et al. [9] showed that early imaging in incarcerated gravid uterus improves timely reduction. Ultrasonographic monitoring before and after reduction is ideal for maternal–fetal assessment, but in this study was precluded by conflict‐related power outages. Clinical evaluation relied on fetoscopic auscultation of fetal heart rate and a thorough maternal examination.

Previous studies have reported multiparity as one of the major factors associated with pelvic organ prolapse [10, 11], and we observed that our patient was also multiparous; however, several other factors could be involved in our case, including the pregnancy she was also carrying [11] and the postnatal course of previous pregnancies, given the low compliance with prenatal consultations and pregnancy follow‐up examinations in rural areas or due to limited resources and very distant hospitals [12].

The Pelvic Organ Prolapse quantification (POP‐Q) classification in that study was not used in this study because the term of pregnancy was not taken into account in it, and other elements may be necessary for the evaluation of the degree of prolapse for our patient, as reported in the study by Deval and Joguet [13]. The length of the uterine cervix, the condition of the uterine cervix, perineal scars, the presence or absence of radial folds of the anus, and the tone of the external sphincter of the anus. We believe that, in the case of prolapse of the pregnant uterus, not only should the classification used take into account the anatomical elements of the pelvic floor, but also the term of pregnancy and soft‐tissue defects, which can help guide management, as shown in the study by Tong et al. [14].

### 3.2. Mechanisms of Uterine Prolapse During Pregnancy

The most frequently reported mechanism in the literature is related to uterine retroversion, a condition relatively common among women. As pregnancy progresses, the uterus typically undergoes spontaneous rotation between the 14th and 16th weeks, assuming an anteverted position that allows the growing uterus to expand within the abdominal cavity [7]. However, this process carries a risk of uterine incarceration.

Risk factors include deep sacral concavity, pelvic adhesions, uterine malformations, and pelvic tumors, more commonly in children and the elderly, but can occur at any age, chronic constipation, severe or chronic cough, pelvic floor dysfunction, and pregnancy [7, 11, 15].

Uterine prolapse into the rectal orifice during pregnancy remains a rare but serious complication, often associated with weakness of the uterosacral ligaments and distension of the pelvic support structures [3]. Recent studies have emphasized the role of biomechanical forces, particularly those arising from connective tissue alterations, increased intra‐abdominal pressure, and straining efforts. In some cases, anatomical defects such as disruption of the rectovaginal septum may also contribute to this pathology [3, 16].

In our patient, not only were these factors likely contributors to the prolapse, but the increased uterine weight associated with pregnancy and hormonal changes may have played a critical role. Furthermore, the biomechanical impact of rural field labor performed by the patient may have exacerbated the risk, highlighting the influence of environmental and occupational stressors on pelvic floor integrity.

### 3.3. Treatment Method and Follow‐Up

It is generally an emergency, as it was suggested that prolapse of the rectum and uterus cannot be reduced spontaneously or manually. The inability to return to a normal position leads to other serious complications, with the risk of fetal loss, strangulation of the uterus and rectum, and so forth [1] That is why, for our patient, given the difficulties linked to resources, the treatment was early and well conducted.

The therapeutic approach described by Hassanin et al. [8] attempted manual reduction of the prolapsed uterus via the anal route under general anesthesia, but sustained pressure failed. Two ring forceps were then applied to the cervix for continuous traction, followed by intrarectal packing to prevent recurrence. The course was complicated by a miscarriage 1 week later.

Existing literature indicates that management depends on gestational age at diagnosis, balancing risks and benefits. In the rare event of a term uterine prolapse, we suggest that vaginal cesarean delivery may safely extract the fetus, followed by reduction akin to that used for incarcerated uteri to restore uterine polarity [14], but in our study, we have seen that for pregnancies not at term with the uterus protruding through the anal orifice, laparotomy is the best option; progressive perineal reduction combined with controlled progressive abdominal reduction could help preserve the pregnancy, especially as its association with anal cerclage can prevent recurrence and thus temporarily restore the polarity of the gravid uterus to achieve full‐term pregnancy. This may be the therapeutic alternative for similar cases in resource‐limited or conflict‐ridden settings.

Nevertheless, surgical repair of the prolapse after childbirth should be discussed in light of the functional discomfort caused and the desire for subsequent pregnancy [17], and intraoperative sacro‐fixation was not performed in view of the pregnancy, so a possible sacro‐fixation of the uterus was proposed to the patient as soon as she gave birth.

### 3.4. The Strengths and Weaknesses of the Study

The strength of this study is as follows: Firstly, the association of uterine incarceration and rectal prolapse during pregnancy is exceptionally rare, and this is among the few documented cases worldwide. Secondly, this case suggests a pragmatic, stepwise approach to surgical management, emphasizing adaptability in a low‐resource environment while maintaining maternal and fetal safety. It demonstrates that laparotomy and anal cerclage can be performed in the low‐resource environment of pregnant women with prolapse to allow a term pregnancy and sacrospinal fixation after delivery.

The weakness of this study relates to the particular case, which is also its limitation, as it cannot draw general conclusions. However, this case reports enough of the data needed in the follow‐up of a prolapsed gravid uterus incarcerated through the anal orifice, and randomized clinical studies can be carried out to give a standard protocol in the face of a prolapsed gravid uterus, incarcerated or not, depending on the term of pregnancy, to enrich this present study.

Key take‐home points:

• The protrusion of an incarcerated gravid uterus through the anal orifice is an exceptionally rare occurrence in clinical practice.
• The presence of an irreducible, painful mass at the anal opening in a pregnant woman, accompanied by a sudden reduction in abdominal volume, may be indicative of an incarcerated uterine prolapse.
• In resource‐limited settings, where urgent perineal ultrasonography is unavailable, pregnancy can be confirmed by a simple test, and auscultation of fetal heart sounds directly over the incarcerated uterus may provide evidence of fetal viability.
• In this extreme emergency, performing a two‐handed reduction along with a laparotomy if manual reduction under relaxation fails can lead to favorable outcomes and prognosis.

## 4. Conclusion

Prolapse of the gravid uterus incarcerated through the anus is a rare pathology that must be managed and treated clinically based on gestational age. However, in resource‐limited settings, stepwise reduction of the gravid uterus by laparotomy, combined with anal cerclage, can temporarily restore the polarity of the gravid uterus, allowing the pregnancy to advance to sacro‐fixation or uterine fixation after delivery.

## Author Contributions

Matabishi Bandeke Destin contributed to the conception, design, assembly of intellectual content, and revision of the case report. Mongwa Mbikilile Justin contributed to both the process and the manuscript′s revision, including the report′s conception. Heritier Balirwa contributed to the design. Byabene Gloire, Mukanire Ntakwinja, and Alumeti Munyali Désiré provided supervision and guidance.

## Funding

No funding was received for this manuscript.

## Ethics Statement

This case was reported following local legislation and institutional guidelines. The study was performed by the principles of the Declaration of Helsinki, and no ethics committee was formed.

## Consent

The patient consented to this publication.

## Conflicts of Interest

The authors declare no conflicts of interest.

## Data Availability

The data that support the findings of this study are available from the corresponding author upon reasonable request.
